# Comparative effects of psychotropic medications on sleep architecture: a retrospective review of diagnostic polysomnography sleep parameters

**DOI:** 10.5935/1984-0063.20200071

**Published:** 2021

**Authors:** Elias Ghossoub, Luna Geagea, Firas Kobeissy, Farid Talih

**Affiliations:** 1 American University of Beirut, Psychiatry, Beirut, Lebanon.; 2 American University of Beirut, Biochemistry and Molecular Genetics, Beirut, Beriut, Lebanon.

**Keywords:** Sleep Wake Disorders, Nocturnal Myoclonus Syndrome, Periodic Limb Movements Disorder, Sleep Apnea

## Abstract

**Objective:**

To study the effects of different psychotropic drugs on sleep architecture and sleep-related disorders.

**Material and Methods:**

In this retrospective review of 405 consecutive de-identiﬁed diagnostic polysomnograms performed at a sleep laboratory from 2007 until 2011, we grouped 347 polysomnograms into ﬁve categories: controls, antidepressants (AD), antidepressants + anticonvulsants (ADAC), antidepressants + antipsychotics (ADAP), antidepressants + anticonvulsants + antipsychotics (ADACP). We conducted pairwise comparisons for demographic characteristics, medical history, speciﬁc psychotropic medication uses and sleep architecture variables, and adjusted for multiple testing. We used logistic regression to determine the odds ratio of having elevated apnea-hypopnea index (AHI) and periodic limb movement index (PLMI) within each group as compared to controls.

**Results:**

Compared to controls, all groups had a signiﬁcantly higher prevalence of benzodiazepines and trazodone use. AD and ADACP had signiﬁcantly longer REM latency and lower REM percentage of total sleep time compared to controls. ADAP had a signiﬁcantly lower AHI compared to controls, but that association was lost in the regression model. AD was associated with a higher PLMI compared to controls.

**Conclusion:**

Psychotropic polypharmacy does not seem to be associated with significantly deleterious effects on sleep architecture. Adjunct anticonvulsants or antipsychotics to antidepressants may protect against periodic limb movement disorder.

## INTRODUCTION

Sleep is defined as “a rapidly reversible state of reduced responsiveness, reduced motor activity and reduced metabolism”^1^. Although the function of sleep is not completely elucidated; it is postulated that it might be an adaptive state that enhances cognition and an individual’s overall functionality when awake1. From this perspective, we can understand the burden of sleep disturbances on one’s functioning and well-being: people with insomnia report fatigue, decreased energy, confusion, and tension^2^. Insomnia is one of the most common patient complaints and was found to account for around five million outpatient visits per year in the United States of America between 1999 and 2010^3^. It is estimated that up to one third of the general population reports at least one criterion of insomnia as defined by the Diagnostic and Statistical Manual of Mental Disorders - 4^th^ edition (DSM-IV)^4^, while 6% of the general population met criteria for a diagnosis of insomnia^5^. Insomnia comorbid with a psychiatric disorder is the most common diagnosis for patients presenting to a sleep center, with a reported prevalence of 3% in the general population^6^. The relationship between sleep disturbances and psychiatric disorders is complex. The latest changes implemented in the 5^th^ edition of the Diagnostic and Statistical Manual (DSM-V) regarding the diagnosis of primary insomnia disorders, suggest that the medical community is increasingly cognizant of a bidirectional relationship between sleep disturbances such as insomnia and excessive daytime sleepiness (EDS) and psychiatric disorders, most notably depression and anxiety^7^.

Not only are psychiatric diseases associated with sleep disturbances, but psychotropic drug treatments can also affect the sleep-wake cycle. Insomnia and EDS are highly comorbid with depression, anxiety and schizophrenia, but part of the problem lays in the use of antidepressants and anxiolytic agents^8^. Antipsychotic and anticonvulsant drugs have also been reported to have sedating effects^9^. Psychotropic drugs can also cause polysomnographic changes, but studies have been few and limited. A recent review found that only paroxetine, doxepin, trimipramine, and trazodone have been studied as treatment options for patients with primary insomnia and their effects on sleep have been evaluated by polysomnography (PSG) with mixed results^10^. Most of the drugs were evaluated in healthy individuals: most antidepressants (except bupropion) have been found to suppress rapid eye movement (REM)^9^ and lithium, trazodone and some antipsychotics have been shown to increase slow wave sleep (SWS)^9,10^. Additionally, the onset and exacerbation of primary sleep disorders such as obstructive sleep apnea (OSA) and periodic limb movement disorder (PLMD) have been associated with the use of a majority of antidepressants and antipsychotics through direct and indirect effects^9,11^.

To the best of our knowledge, the comparative effects of psychotropic polypharmacy on sleep architecture and sleep-related disorders in a sizable PSG database has not been examined. The primary objective of this study is to compare sleep architecture as detected by PSGs between groups of participants: participants on antidepressants (AD), participants on combinations of antidepressants and anticonvulsants (ADAC), participants on combinations of antidepressants and antipsychotics (ADAP), participants on combinations of antidepressants and anticonvulsants and antipsychotics (ADACP), and controls (C). The secondary objective of this study is to compare the prevalence of sleep disorders as detected by PSG across groups of subjects taking different psychotropics. The sleep disorders to be examined will be obstructive sleep apnea (OSA) and periodic limb movement disorder (PLMD).

## MATERIAL AND METHODS

### Data source and collection

 The study is a retrospective review of de-identified diagnostic PSG reports. PSGs were conducted at a hospital-based sleep disorders center in Ohio, from 2007 until 2011. The sleep tests were conducted under the supervision of a board-certified sleep medicine physician (psychiatrist) as per the protocols of the American Academy of Sleep Medicine (AASM) for diagnostic PSG and the AASM Manual for the Scoring of Sleep and Associated Events^12^. The sleep studies at the above-mentioned sleep lab were initially scored by a registered polysomnographic technologist (RGPST) and subsequently reviewed, validated, and finalized by a physician certified in sleep medicine. The physician was also responsible for generating the final PSG reports. Data for each individual PSG was retrieved from de-identified sleep study electronic database. We included all diagnostic PSGs performed on adults (18 years or older). We excluded split night studies and PSGs that were conducted under supplemental oxygen or for treatment purposes (such as those conducted with positive airway pressure).

### Ethical considerations

This study was approved by the Institutional Review Board (IRB) at the American University of Beirut (AUB) and has been performed in accordance with the ethical standards laid down in the Helsinki II Declaration about anonymity. Given that this study involved only existing de-identified records it was exempted from full review by the AUB IRB, and no informed consent was required of the patients.

### Measures

#### Demographic and clinical data

We retrieved the following information for each participant: age, sex, neck circumference (NC), body mass index (BMI), tobacco, alcohol and caffeine use, and Epworth sleepiness scale (ESS) scores. The ESS is an eight-item self-administered questionnaire that measures sleepiness in eight daytime activities, each on a four-point scale (scored from 0 to 3); scores above 10 were significantly correlated with EDS^13^. We also recorded medical history as noted in the participant’s chart, including any psychiatric, cardiovascular, respiratory, neurological, metabolic disorders, and pain symptoms.

### Current psychotropic medication intake

Each participant’s medications were documented in the PSG report. We recorded the psychotropic use as per the following classes: selective serotonin reuptake inhibitors (SSRI), serotonin-norepinephrine reuptake inhibitors (SNRI), tricyclic antidepressants (TCA), bupropion, mirtazapine, trazodone, benzodiazepines, hypnotics, lithium, anticonvulsants (AC), antipsychotics (AP), opiates, and antihistamines.

For statistical purposes, we computed our main variable of interest (D) based on the above. The variable (D) has the following categories:

1. **Antidepressants (AD) group** includes participants who were on SSRI and/or SNRI and/or TCA. We grouped these three types of common antidepressants together because they have similar mechanisms of action^14^;
2. **Antidepressants and Anticonvulsants (ADAC) group** includes participants who were on a combination of: (a) SSRI and/or SNRI and/or TCA; and (b) AC;
3. **Antidepressants and Antipsychotics (ADAP) group** includes participants who were on a combination of: (a) SSRI and/or SNRI and/or TCA; and (b) AP;
4. **Antidepressants and Anticonvulsants and Antipsychotics (ADACP) group** includes participants who were on a combination of: (a) SSRI and/or SNRI and/or TCA; and (b) AC and AP;
5. **Controls (C) group** includes the remainder of the participants, who were not on SSRI, SNRI, TCA, AC or AP.

### Sleep architecture parameters examined

The sleep architecture variables included, as defined by the International Classification of Sleep Disorders (ICSD-2)^15^ are: time in bed (TIB), total sleep period (TSP), total sleep time (TST), sleep efficiency (SE), sleep onset latency (SOL), number of REM periods, REM onset latency, wake after sleep onset (WASO), number of awakenings, total arousal index (ARI), apnea-hypopnea index (AHI), periodic limb movement index (PLMI), minimal oxygen saturation during sleep, mean heart rate (HR) in non-REM (NREM) sleep, and sleep stages distribution.

### Sleep disorders related variables (OSA and PLMD)

Our first dependent variable is the measure of a significant AHI, which is one of the main diagnostic criteria of OSA^16^. We computed a dichotomous variable to measure the presence of OSA based on the AHI cut-off of 5/hour^16,17^. Participants who had an AHI equal or greater to 5 were deemed to have a “raised AHI”.

Our second dependent variable is the measure of a significantly elevated PLMI, one of the main diagnostic criteria of periodic limb movement disorder (PLMD)^16^. We also computed a dichotomous variable to measure the presence of PLMD based on the PLMI cut-off of 15/hour^16,18^. Participants who had a PLMI equal or greater to 15 were considered to have a “high PLMI”.

### Statistical analysis plan

First, we compared demographic, psychotropic medication intake and sleep architecture variables, across the 5 groups C, AD, ADAC, ADAP and ADACP of our variable of interest (D). We used Kruskal-Wallis tests for continuous variables and Chi-square test for dichotomous variables. We then conducted bivariate analyses for (D) and the other variables on both of our outcome variables. We measured the associations with the Mann-Whitney test for continuous variables and Chi-square test for dichotomous variables. For both sets of bivariate analyses, we determined statistical significance using two-sided tests at the alpha level cut-off of 5% and we used Bonferroni’s correction method to adjust for multiple testing. Finally, we entered (D) in two binary logistic regression models on the OSA and the PLMD outcome variables. We adjusted both models according to the set of control variables, including socio-demographic characteristics, sleep architecture parameters and concomitant medications used such as benzodiazepines and trazodone, which were significant in the bivariate analyses. We selected the models that best fitted the data using a forward stepwise approach in order to calculate the adjusted odds ratios (aOR) and their corresponding 95% confidence intervals (95%CI) of having “raised AHI” or “high PLMI” on taking psychotropics. We finally did a sensitivity analysis in which we raised the cut-off to determine “raised AHI” to 10/hour and 15/hour, to analyze whether the associations with the use of psychotropics would change. We used IBM’s Statistical Package for the Social Sciences (SPSS) version 21 to conduct the data analysis.

## RESULTS

### Sample characteristics

**Figure 1:**
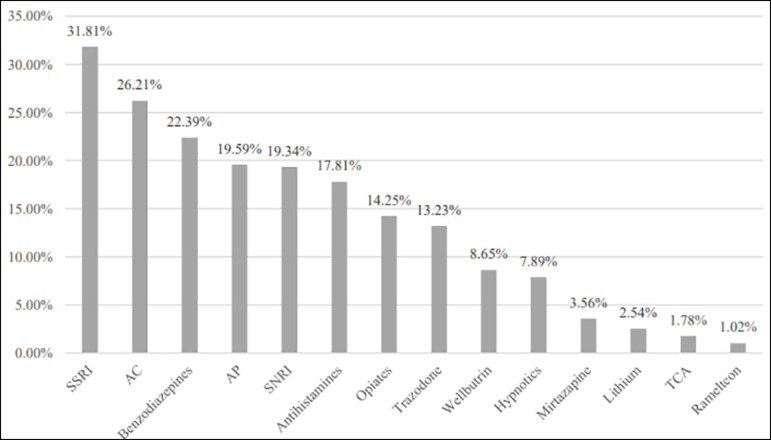
Frequency in percent of psychotropic drug intake in the total sample.

The total sample (N=405) included participants aged 18 to 92 years old (mean age: 45.75 years) with an average BMI of 34.50 and an average neck circumference of 40.41 cm. Males constituted around 40% of the sample. More than 40% of participants reported smoking tobacco and more than 80% reported drinking caffeinated beverages. Significant proportions of our sample had medical comorbidities: 76.38% had a psychiatric disorder, 54.27% had cardiovascular disease and 66.58% reported having pain symptoms. As detailed in Figure 1, around one-third of our sample was taking an SSRI, one-quarter was taking an AC, and one-fifth was taking an AP.

 From the 405 records obtained, only 347 participants (85.68%) had valid data to be included in (D): 105 (valid percent: 30.26%) were classified as AD, 40 (11.53%) as ADAC, 28 (8.07%) as ADAP and 28 (8.07%) as ADACP. Compared to C, all D groups had significantly higher proportions of participants who had psychiatric disorders (*p*<0.001) and who used benzodiazepines (*p*<0.001) and trazodone (*p*<0.001). Participants in AD and ADAC had lower proportions of males. Detailed data is presented in Supplementary Table 1.

### Comparison of sleep architecture parameters across (D) categories

As shown in Table 1, there were significant differences in TST (*p*=0.002), SE (*p*=0.001), REM onset latency (*p*<0.001), WASO (*p*<0.001), total arousal index (*p*=0.002), AHI (*p*=0.009) and PLMI (*p*=0.042) across the five groups. The AD and ADACP groups had a significantly more delayed REM onset (*p*<0.001 and *p*=0.007, respectively) and a shorter REM stage (*p*=0.001 and *p*=.0.009, respectively) compared to C. The ADAP group had a better SE (*p*=0.003) and a shorter WASO (*p*=0.001) compared to controls. Furthermore, the total arousal index was significantly lower among ADAP participants compared to C (*p*=0.027) and AD (*p*=0.026) participants.

**Table 1.** Comparison of sleep architecture parameters according to psychotropic drug intake groups.

Parameter	C (N=146;P=42.08%)	AD (N=105;P=30.26%)	ADAC (N=40;P=11.53%)	ADAP (N=28;P=8.07%)	ADACP (N=28;P=8.07%)	p
	Mean (SD)	Mean (SD)	Mean (SD)	Mean (SD)	Mean (SD)	
**TIB (in minutes)**	418.05 (44.83)	413.34 (49.00)	418.53 (36.03)	440.82 (59.04)	415.68 (33.66)	0.232
**TSP (in minutes)**	384.72 (58.99)	374.23 (71.64)	378.53 (69.95)	405.29 (63.37)	364.95 (94.29)	0.223
**TST (in minutes)**	309.95 (81.90)	327.34 (84.14)	334.61 (79.35)	**370.71 (74.94)**	326.07 (101.79)	0.002
**SE (in %)**	73.85 (17.44)	78.65 (18.40)	79.74 (16.94)	**84.31 (13.85)**	78.01 (22.75)	0.001
**SOL (in minutes)**	28.03 (28.43)	31.81 (31.93)	36.54 (54.83)	31.41 (36.88)	36.45 (48.68)	0.868
**Number of REM periods**	6.62(5.40)	**3.98 (3.50)**	**4.15 (4.02)**	5.71 (5.72)	**3.18 (3.55)**	<0.001
**REM onset latency (in minutes)**	151.94 (81.38)	**209.19 (88.81)**	189.84 (97.60)	169.28 (90.47)	**218.21 (84.22)**	<0.001
**WASO (in minutes)**	79.77 (65.23)	**54.15 (54.10**	**47.31 (39.73)**	**38.61 (46.74)**	**49.84 (56.73)**	<0.001
**Total arousal index**	19.19 (16.33)	20.21 (17.12)	15.53 (11.79	15.08 * (19.39)	13.12 (12.44)	0.002
**AHI**	17.31 (20.77)	13.94 (18.44)	9.98 (14.67)	**9.37 (17.50)**	9.37 (11.84)	0.009
**PLMI**	19.44 (24.25	30.73 * (39.32)	22.07 (26.06)	17.91 (15.25)	17.59 (32.81)	0.042
**Minimal SaO2 (in %)**	82.65 (11.70)	84.58 (8.29	86.38 (5.17)	86.21 (9.04)	83.86 (9.55)	0.168
**HR in NREM sleep**	67.41 (9.87)	**71.77 (11.29)**	**74.61 (11.37)**	71.97 (15.25)	**80.26 (14.32)**	<0.001
**Sleep stage distribution(in percent)**						
**Stage 1**	15.82 (12.98)	13.93 (10.56)	10.66 (6.53)	8.32 * (4.80)	10.28 (7.23)	<0.001
**Stage 2**	54.67 (12.53)	**60.19 (12.26)**	55.60 (16.74)	60.45 (15.29)	**63.05 (14.35)**	0.001
**SWS**	16.12 (10.79)	15.89 (10.99)	21.66 (15.25)	19.01 (16.39)	18.85 (13.76)	0.286
**REM**	13.40 (7.22)	**10.01 (7.54)**	12.08 (10.36)	12.20 (8.05)	**8.27 (6.31)**	<0.001

Kruskal-Wallis test was used for the analyses of continuous variables. Pearson’s Chi-Square test was used for the analyses of categorical variables. **Bold:** significantly different than C after adjusting for multiple comparisons.

* significantly different than AD after adjusting for multiple comparisons

**Abbreviations:** C: controls group; AD: antidepressants group; ADACP: antidepressants and anticonvulsants and/or antipsychotics group; N: count; P: valid percent; SD: standard deviation; TIB: time in bed; TSP: total sleep period; TST: total sleep time; SE: sleep efficiency; SOL: sleep onset latency; REM: rapid eye movement; WASO: wake after sleep onset; AHI: apnea-hypopnea index; PLMI: periodic limb movement index; SaO2: oxygen saturation; HR: heart rate; SWS: slow-wave sleep

### Characteristics and sleep architecture parameters among participants with raised AHI

Based on the criteria we described in the methodology section; 242 out of 405 participants (59.75%) were classified as having “raised AHI”. As detailed in Supplementary Table 2, this group of participants was significantly older (49.08 years vs. 40.80 years; *p*<0.001), more obese (36.47 vs. 31.56; *p*<0.001) and included more males (47.52% vs. 30.67%; *p*=0.001) as compared to the “normal AHI” group. The comparison of sleep architecture parameters is presented in Table 2. Surprisingly, there were no differences in SE (77.44% vs. 78.51%; *p*=0.101) between the two groups. The “raised AHI” group had a significantly longer WASO (*p*<0.001) and shorter SWS sleep stage (*p*<0.001).

**Table 2.** Comparison of sleep architecture parameters according to diagnosis of obstructive sleep apnea (on the left) and periodic limb movement disorder (on the right).

Parameter	Raised AHI (N=242; P=59.75%)	Normal AHI (N=163; P=40.45%)	p	High PLMI (N=165; P=40.74%)	Normal PLMI (N=240; P=59.51%)	p
	Mean (SD)	Mean (SD)		Mean (SD)	Mean (SD)	
**TIB (in minutes)**	418.29 (45.16)	421.20 (50.69)	0.876	413.34 (49.00)	418.05 (44.83)	0.435
**TSP (in minutes)**	386.01 (66.10)	378.26 (73.14)	0.151	374.23 (71.64)	384.72 (58.99)	0.274
**TST (in minutes)**	325.37 (80.28)	332.73 (91.82)	0.355	313.96 (84.21)	338.21 (84.44)	0.002
**SE (in %)**	77.44 (16.92)	78.51 (18.81)	0.101	74.86 (18.52)	79.94 (16.82)	0.001
**SOL (in minutes)**	26.42 (28.25)	35.67 (42.64)	0.118	33.00 (40.97)	28.19 (30.18)	0.338
**REM onset Latency (in minutes)**	178.63 (92.07)	163.46 (81.91)	0.213	180.56 (83.58)	167.09 (91.09)	0.055
**WASO (in minutes)**	65.90 (57.54)	52.78 (56.22)	<0.001	69.97 (61.50)	54.19 (53.42)	0.001
**Number of awakenings**	25.58 (20.25)	18.21 (13.17)	0.001	21.92 (18.22)	25.08 (18.12)	<0.001
**Number of REM periods**	5.61 (5.11)	4.93 (4.09)	0.542	5.15 (5.01)	5.47 (4.55)	0.223
**Total arousal index**	21.82 (18.51)	13.16 (9.83)	<0.001	23.25 (16.84	14.95 (14.79)	<0.001
**AHI**	22.33 (20.57)	1.51 (1.40)	<0.001	16.65 (19.34)	12.09 (18.42)	0.002
**PLMI**	23.96 (30.72)	19.44 (30.25)	0.012	47.62 (34.24)	4.63 (4.45)	<0.001
**Minimal SaO2 (in %)**	80.05 (10.40)	89.94 (3.56)	<0.001	84.58 (8.29)	82.65 (11.70)	0.465
**HR in NREM Sleep**	71.11 (12.33)	70.37 (10.84)	0.949	70.67 (10.96)	70.91 (12.28)	0.851
**Sleep stagedistribution (in percent)**						
**Stage 1**	14.88 (11.83)	11.12 (9.62)	<0.001	16.11 (13.06)	11.48 (9.16)	<0.001
**Stage 2**	57.54 (13.62)	55.95 (13.60)	0.153	55.13 (14.36)	58.12 (12.98)	0.067
**SWS**	15.66 (11.63)	20.24 (12.53)	<0.001	17.07 (12.35)	17.81 (12.10)	0.594
REM	11.92 (7.65)	12.77 (8.18)	0.341	11.71 (8.14)	12.64 (7.67)	0.117

**Notes:** Mann-Whitney test was used for the analyses. **Abbreviations: ** AHI: Apnea-hypopnea index; PLMI: Periodic limb movement index; N: Count; P: Valid

### Characteristics and sleep architecture parameters among participants with high PLMI

Among our total sample, 165 participants (40.74%) were classified as having a “high PLMI”. This group was significantly older (49.70 years vs. 43.03 years; *p*<0.001) than the “normal PLMI” group. A significantly lower proportion of participants in the “high PLMI” group were using benzodiazepines (15.63% vs. 27.04%; *p*=0.008); detailed results are provided in Supplementary Table 2. As shown in Table 2, the “high PLMI” group had a lower SE (74.86% vs. 79.94%; *p*=0.001) and higher AHI (16.65 vs. 12.09; *p*=0.002). Overall, the REM sleep stage parameters were similar between the two groups.

### Association of (D) with raised AHI and high PLMI

Around 60% of the “raised AHI” group were classified as either AD, ADAC, ADAP or ADACP. Table 3 shows that participants in the ADAP and ADACP groups were significantly less likely to have a raised AHI when compared to controls OR=0.40; 95%CI=(0.18-0.91). After adjusting for relevant confounders such as sex and BMI, the significance of the associations with ADAP [aOR=0.74; 95%CI=(0.20-2.78)] and [ADACP aOR=0.24; 95%CI=(0.05-1.05)] were lost. Our sensitivity analyses (with AHI cut-offs of 10/hour and 15/hour) did not yield significant associations between any of the groups and having an elevated AHI (data not shown).

**Table 3.** Odds ratios from bivariate logistic regression analyses of having a high apnea-hypopnea index (on the left) and a high periodic limb movement index (on the right) on psychotropic drug intake.

Psychotropic drug intake (D)	Raised AHI Unadjusted model OR (95%CI)	Best-fit model aOR (95%CI)	High PLMI Unadjusted model OR (95%CI)	Best-fit model aOR (95%CI)
**C (reference)**	1.00	1.00	1.00	1.00
**AD**	0.66 (0.39-1.12)	0.65 (0.27-1.59)	1.58 (0.95-2.61)	**2.08 (1.17-3.70)**
**ADAC**	0.69 (0.34-1.42)	1.66 (0.54-5.06)	0.86 (0.42-1.77)	1.21 (0.56-2.62)
**ADAP**	**0.40 (0.18-0.91)**	0.74 (0.20-2.78)	0.57 (0.24-1.39)	0.94 (0.35-2.49)
**ADACP**	**0.40 (0.18-0.91)**	0.24 (0.05-1.05)	0.39 (0.15-1.02)	0.71 (0.25-2.03)

**Notes:** AHI: Apnea-hypopnea index; PLMI: Periodic limb movement index; OR: Odds ratio; aOR: Adjusted odds ratio; 95%CI: 95% Confidence interval. **Abbreviations: C: ** Controls group; AD: Antidepressants group; ADAC: Antidepressants and anticonvulsants;ADAP: Antidepressants and antipsychotics; ADACP: Antidepressants and anticonvulsants and antipsychotics group; Bold: Significantly different than C at p<0.05.

Close to 60% of the high PLMI group were classified as AD, ADAC, ADAP or ADACP. As seen in supplementary table 2, participants in the AD group were significantly more likely to have a high PLMI when compared to controls [aOR=2.08; 95%CI=(1.17-3.70)] after adjusting for confounders such as age and benzodiazepine use. All other groups were not significantly associated with a high PLMI.

## DISCUSSION

The use of common psychotropic drugs such as antidepressants, anticonvulsants and antipsychotics was found to be associated with changes in sleep architecture in a sample of patients with disturbed sleep presenting for polysomnography. Overall, AD use was associated with reduced REM, while ADAP alone was associated with improved sleep efficiency and decreased arousal. The use of these psychotropics was not associated with higher odds of having a raised AHI. On the other hand, AD use was associated with higher odds of having a high PLMI whereas ADAC, ADAP and ADACP use were not.

The findings of our study highlight the bidirectional relationship between sleep and psychiatric disorders: around 76% of those presenting for sleep testing had some type of psychiatric disorder per their medical record. Taking psychotropic drugs seems to have a beneficial effect on sleep architecture: all groups had shorter wake periods after sleep onset, while ADAP had a better sleep efficiency (SE). These findings are particularly important given that nearly all psychiatric disorders are associated with worsening SE^19^. Both AD and ADACP groups had longer REM latency and shorter REM periods. The findings of our study are in line with previous research, which found that monoaminergic antidepressants suppress REM sleep^20^. Furthermore, as per our analyses, the effects of SSRI, SNRI and TCA on REM sleep did not seem to be affected by the addition of anticonvulsants but seem to have been countered by adding antipsychotics.

Being in the AD, ADAC, ADAP or ADACP groups did not seem to increase the risk of having a high AHI, regardless of the cut-off used. This was an interesting finding, given that most psychotropic drugs, especially antipsychotics, are associated with weight gain^21^, which is a major risk factor for OSA^22^. Additionally, all groups, including controls, had similar BMI and NC profiles. All of this suggests that psychotropic drug intake does not substantially increase the risk of having OSA in a population presenting for diagnostic PSG. Conversely, the AD group appeared to have a worse PLMI and be more at risk for PLMD, which is consistent with the literature^11,23^. However, it is unclear why the ADAC, ADAP and ADACP groups would have less PLMI. While it is possible that anticonvulsants antagonize the effect of SSRI, SNRI and TCA on periodic leg movements, antipsychotics have been found to potentially worsen periodic leg movements and clinically exacerbate restless legs syndrome due to dopaminergic antagonism^21^.

There are several limitations to our study. First, although our sample size was large, we had small samples on combinations of antidepressants and anticonvulsants or antidepressants and antipsychotics, which might have affected the accuracy of our results. In addition, 46.5% of the control group had a medical history of psychiatric disorders, which makes this group non-homogeneous. Future studies can investigate the implications of having untreated psychiatric disorders on sleep architecture. Second, we did not have information about the participants’ medication indication, adherence and dosages; such information is highly relevant because medication sleep effects might be dose-dependent^21^. Third, our diagnoses of OSA and PLMD were solely based on PSG interpretation and did not include clinical criteria; however, it is unlikely that this limitation has significantly affected the accuracy of our results given that PSG are considered a requirement to establish both diagnoses^24,25^. Fourth, we controlled for the presence of psychiatric disorders by relying on information provided by the participants and documented in the PSG reports, and not on structured clinical interviews. Since this information was self-reported and was not sufficiently reliable, we were unable to control for specific psychiatric diagnoses in our analyses. Fifth, our study’s population is restricted to individuals referred for PSG due to sleeping difficulties and therefore our results might not be generalizable to the general population; however, to the best of our knowledge, our study is the first to address the association of polypharmacy with sleep architecture in a real-world setting. Finally, since our study design is a retrospective review, we are unable to establish a causal relationship between psychotropic drug intakes and sleep architecture disturbances or developing OSA or PLMD.

Our results seem to show that it is unlikely that exposure to psychotropics polymedication has a significantly deleterious effect on overall sleep architecture among patients presenting for PSGs. We did not find an association between exposure to psychotropics and significantly elevated AHI. However, it is possible that exposure to psychotropics contributes over time towards developing OSA through different mechanisms, including weight gain. Future research should look into a causal relationship between psychotropics and OSA through prospective longitudinal studies. Our findings also highlight the potential benefit of adjuvant anticonvulsants to antidepressants in decreasing periodic limb movements during sleep. Given that currently there are no evidence-based guidelines for management of PLMD^26^, treatment with anticonvulsants can be a potential avenue for future research.

## Supplementary Material

**Supplementary Table 1:** Distribution of sample characteristics according to psychotropic drug intake groups

Characteristic	C (N=146;P=42.08%)	AD (N=105;P=30.26%)	ADAC (N=40; P=11.53%)	ADAP (N=28; P=8.07%)	ADACP (N=28; P=8.07%)	p
	Mean (SD)	Mean (SD)	Mean (SD)	Mean (SD)	Mean (SD)	
**Age (in years)**	48.09 (14.30)	46.97 (14.78)	45.80 (12.71)	40.29 (13.53)	42.50 (9.78)	0.091
**NC (in cm)**	40.97 (5.00)	39.47 (4.45)	40.39 (4.83)	40.06 (4.75)	41.99 (4.11)	0.031
**BMI**	33.87 (8.00)	34.01 (7.52)	37.39 (8.95)	32.96 (6.98)	37.02 (7.96)	0.052
**ESS Score**	10.27 (5.36)	10.65 (5.34)	11.48 (4.67)	12.07 (5.82)	11.79 (5.60)	0.229
	N (P)	N (P)	N (P)	N (P)	N (P)	
**Male Sex**	84 (57.53)	**28 (26.67)**	**9 (22.50)**	10 (35.71)	10 (35.71)	<0.001
**Tobacco Use**	44 (30.77)	44 (41.90)	17 (42.50)	15 (55.56)	14 (56.00)	0.030
**Alcohol Use**	24 (16.67)	12 (11.43)	2 (5.00)	3 (11.11)	2 (8.00)	0.345^a^
**Caffeine Use**	115 (80.99)	90 (88.24)	29 (76.32)	16 (61.54)	22 (91.67)	0.018^a^
**Medical History**						
**Psychiatric**	66 (46.48)	**102 (97.14)**	**40 (100.00)**	**28 (100.00)**	**28 (100.00)**	<0.001
**Cardiovascular**	83 (58.45)	59 (56.19)	19 (47.50)	12 (42.86)	17 (60.71)	0.452
**Respiratory**	30 (21.13)	36 (34.29)	13 (32.50)	3 (10.71)	10 (35.71)	0.029
**Neurological**	13 (9.15)	11 (10.48)	**12 (30.00)**	1 (3.57)	**8 (28.57)**	0.001^a^
**Pain**	75 (52.82)	**76 (72.38)**	**35 (87.50)**	21 (75.00)	20 (71.43)	<0.001
**Other Psychotropic Drug Intake**						
**Benzodiazepines**	12 (8.22)	**35 (33.33)**	**10 (25.00)**	**8 (28.57)**	**12 (42.86)**	<0.001
**Hypnotics**	5 (3.42)	13 (12.38)	4 (10.00)	2 (7.14)	4 (14.29)	0.037^a^
**Wellbutrin**	7 (4.79)	12 (11.43)	3 (7.50)	2 (7.14)	1 (3.57)	0.346^a^
**Trazodone**	5 (3.42)	**17 (16.19)**	**9 (22.50)**	**7 (25.00)**	**10 (35.71)**	<0.001^a^
**Mirtazapine**	3 (2.05)	2 (1.90)	4 (10.00)	2 (7.14)	1 (3.57)	0.078^a^
**Lithium**	1 (0.68)	2 (1.90)	2 (5.00)	1 (3.57)	2 (7.14)	0.080^a^
**Opiates**	13 (8.90)	13 (12.38)	10 (25.00)	4 (14.29)	3 (10.71)	0.121^a^
**Antihistamines**	17 (11.64)	21 (20.00)	9 (22.50)	9 (32.14)	7 (25.00)	0.050

Notes: Kruskal-Wallis test was used for the analyses of continuous variables. Pearson’s Chi-Square test was used for the analyses of categorical variables, except where marked with a, which refers to the use of Fisher’s exact test.

**Bold:** significantly different than C after adjusting for multiple comparisons

C: controls group; AD: antidepressants group; ADACP: antidepressants and anticonvulsants and/or antipsychotics group; N: count; P: valid percent; SD: standard deviation; NC: neck circumference; BMI: body mass index; ESS: Epworth sleepiness scale

**Supplementary Table 2:** Distribution of sample characteristics according to diagnoses of obstructive sleep apnea (on the left) and periodic limb movement disorder (on the right)

Characteristic	Raised AHI (N=242; P=59.75%)	Normal AHI (N=163; P=40.45%)	p	High PLMI (N=165; P=40.74%)	Normal PLMI (N=240; P=59.51%)	p
	Mean (SD)	Mean (SD)		Mean (SD)	Mean (SD)	
**Age (in years)**	49.08 (13.80)	40.80 (12.75)	<0.001	49.70 (14.37)	43.03 (13.05)	<0.001
**NC (in cm)**	42.04 (4.27)	38.00 (4.22)	<0.001	40.97 (4.75)	40.03 (4.57)	0.104
**BMI**	36.47 (8.10)	31.56 (6.77)	<0.001	34.30 (7.90)	34.64 (8.01)	0.525
**ESS Score**	11.15 (5.24)	10.59 (5.50)	0.344	10.05 (5.46)	11.52 (5.19)	0.004
	N (valid %)	N (valid %)		N (valid %)	N (valid %)	
**Male Sex**	115 (47.52)	50 (30.67)	0.001	74 (44.85)	91 (37.92)	0.163
**Tobacco Use**	89 (37.24)	71 (44.65)	0.139	62 (37.80)	98 (41.88)	0.414
**Alcohol Use**	30 (12.50)	21 (13.21)	0.836	17 (10.37)	34 (14.47)	0.227
**Caffeine Use**	194 (83.26)	127 (81.94)	0.735	134 (82.72)	187 (82.74)	0.994
**Medical History**						
**Psychiatric**	170 (71.73)	134 (83.23)	0.008	123 (75.46)	181 (77.02)	0.718
**Cardiovascular**	148 (62.45)	68 (42.24)	<0.001	102 (62.58)	114 (48.51)	0.006
**Respiratory**	60 (25.32)	47 (29.19)	0.392	42 (25.77)	65 (27.66)	0.675
**Neurological**	28 (11.81)	24 (14.91)	0.369	15 (9.20)	37 (15.74)	0.057
**Pain**	158 (66.67)	107 (66.46)	0.966	106 (65.03)	159 (67.66)	0.585
**Psychotropic Drug Intake (D)**			0.071			0.018
**C**	100 (47.17)	46 (34.07)		60 (41.67)	86 (42.36)	
**AD**	62 (29.25)	43 (31.85)		55 (38.19)	50 (24.63)	
**ADAC**	24 (11.32)	16 (11.85)		15 (10.42)	25 (12.32)	
**ADAP**	13 (6.13)	15 (11.11)		8 (5.56)	20 (9.85)	
**ADACP**	13 (6.13)	15 (11.11)		6 (4.17)	22 (10.84)	
**Other Psychotropic Drug Intake**						
**Benzodiazepines**	53 (22.65)	35 (22.01)	0.882	25 (15.63)	63 (27.04)	0.008
**Hypnotics**	15 (6.41)	16 (10.06)	0.187	15 (9.38)	16 (6.87)	0.365
**Wellbutrin**	21 (8.97)	13 (8.18)	0.782	18 (11.25)	16 (6.87)	0.129
**Trazodone**	35 (14.96)	17 (10.69)	0.221	18 (11.25)	34 (14.59)	0.337
**Mirtazapine**	8 (3.42)	6 (3.77)	0.852	6 (3.75)	8 (3.43)	0.868
**Lithium**	9 (3.85)	1 (0.63)	0.054a	3 (1.88)	7 (3.00)	0.746a
**Opiates**	31 (13.25)	25 (15.72)	0.491	26 (16.25)	30 (12.88)	0.347
**Antihistamines**	46 (19.66)	24 (15.09)	0.246	27 (16.88)	43 (18.45)	0.688

Mann-Whitney test was used for the analyses of continuous variables. Pearson’s Chi-Square test was used for the analyses of categorical variables, except where marked with a, which refers to the use of Fisher’s exact test.

AHI: apnea-hypopnea index; PLMI: periodic limb movement index; N: count; P: valid percent; SD: standard deviation; NC: neck circumference; BMI: body mass index; ESS: Epworth sleepiness scale; C: controls group; AD: antidepressants group; ADAC: antidepressants and anticonvulsants; ADAP: antidepressants and antipsychotics; ADACP: antidepressants and anticonvulsants and antipsychotics group
